# Supplementary material for: Patterns of bird-window collisions inform mitigation on a university campus
Source: PeerJ. 2016 Feb 1;4:e1652. doi: 10.7717/peerj.1652 (PMC4741078; doi:10.7717/peerj.1652)
Supplement: Data S2 [file peerj-04-1652-s002.docx]

**Supplementary data 1.** Newspaper and TV stories about bird-window collisions at Duke University.

| **Date** | **Media** | **Title** | **Link** |
| --- | --- | --- | --- |
| 5/23/2013 | The Chronicle (Duke University’s newspaper) | Grad student advocates for bird-friendly windows | <http://www.dukechronicle.com/article/2013/05/grad-student-advocates-bird-friendly-windows> |
| 4/16/2015 | American Birding Association Blog | Open Mic: Bird-window collisions and “green” buildings on Duke’s campus | <http://blog.aba.org/2015/04/open-mic-bird-window-collisions-and-green-buildings-on-dukes-campus.html> |
| 5/22/2015 | The Chronicle (Duke University’s newspaper) | Duke's bird safety one of worst in nation according to Bird Window Collision Project | <http://www.dukechronicle.com/article/2015/05/dukes-bird-safety-one-worst-nation-according-bird-window-collision-project#.VV8ubFVViko> |
| 6/15/2015 | WNCN (local associate of | Duke University’s bird collision problem | <http://wncn.com/2015/06/15/duke-universitys-bird-collision-problem/> |
| 6/17/2015 | The News & Observer | Duke ‘green’ building blamed for bird deaths | <http://www.newsobserver.com/news/local/community/durham-news/article24691264.html> |
| 6/21/2015 | Glass Magazine Blog | Birds and glass | <http://fromthefabricator.blogspot.com/2015/06/birds-and-glass.html> |
| 6/24/2015 | WRAL | Duke researchers hope to make campus building more bird-friendly | <http://www.wral.com/duke-researchers-hope-to-make-campus-building-more-bird-friendly/14735932/> |
| 7/3/2015 | The News & Observer | Bob Wilson: Duke’s bird killer and the law of unintended consequences | <http://www.newsobserver.com/news/local/community/durham-news/dn-opinion/article25831687.html> |
| 8/25/2015 | The Chronicle (Duke University’s newspaper) | Birds fly free at Duke: CIEMAS adds patterned film | <http://www.dukechronicle.com/article/2015/08/birds-fly-free-at-duke-ciemas-adds-patterned-film> |
| 9/15/2015 | WRAL | Duke adds design to keep birds from flying into building | <http://www.wral.com/duke-adds-design-to-keep-birds-from-flying-into-building/14901036/> |
